# Why we need more research into the placebo response in psychiatry

**DOI:** 10.1017/S0033291720003633

**Published:** 2020-10

**Authors:** Nathan T.M. Huneke, Nic van der Wee, Matthew Garner, David S. Baldwin

**Affiliations:** 1 Clinical and Experimental Sciences, Faculty of Medicine, University of Southampton, Southampton, UK; 2 University Department of Psychiatry, Academic Centre, College Keep, 4-12 Terminus Terrace, Southampton, SO14 3DT, UK; 3 Department of Psychiatry, Leiden University Medical Center, Leiden, The Netherlands; 4 Academic Unit of Psychology, Faculty of Environmental and Life Sciences, University of Southampton, Southampton, UK; 5 University Department of Psychiatry and Mental Health, University of Cape Town, Cape Town, South Africa

**Keywords:** Placebo response, placebo, psychiatric medicine, psychotropic trials

## Abstract

Placebos are not inert, but exert measurable biological effects. The placebo response in psychiatric illness is important and clinically relevant, but remains poorly understood. In this paper, we review current knowledge about the placebo response in psychiatric medicine and identify research directions for the future. We argue that more research is needed into the placebo response in psychiatric medicine for three broad reasons. First, awareness of factors that cause placebo response, for whom, and when, within clinical trials will allow us to better evidence efficacy of new treatments. Second, by understanding how placebo mechanisms operate in the clinic, we can take advantage of these to optimise the effects of current treatments. Finally, exploring the biological mechanisms of placebo effects might reveal tractable targets for novel treatment development.

## Introduction

A placebo is an inert substance or sham procedure given either as a form of psychological reassurance or to act as a control when testing the efficacy of active treatment. However, placebos are not inert in terms of outcomes. Since the advent of the placebo-controlled trial, it has been observed that patients in the placebo arm can show substantial improvements in symptoms (Beecher, 1955; McQueen, Cohen, St John-Smith, & Rampes, 2013). These observed improvements in symptoms are partly explained by non-specific effects, such as regression to the mean, epiphenomena related to the trial, or sampling bias from dropouts of the least improved (Ashar, Chang, & Wager, 2017; Ernst & Resch, 1995; Miller & Rosenstein, 2006). But, these improvements also result from specific placebo effects, which can be measured in a clinical trial by comparing a placebo arm with a ‘natural history’ or untreated arm (Ernst & Resch, 1995). These concepts have recently been captured in operational definitions reached by expert consensus. The ‘placebo response’ is defined as all within-group improvements that occur following administration of an inactive treatment and is attributable to both non-specific effects such as spontaneous improvement and specific placebo mechanisms (Evers et al., 2018). By contrast, the ‘placebo effect’ is the symptom improvement that is attributable to placebo mechanisms only (Evers et al., 2018). The placebo effect results from an interplay between expectations and learning that causes changes in biological systems including the immune system, hypothalamic–pituitary–adrenal axis and the endogenous opioid system (Benedetti, Carlino, & Pollo, 2011; Evers et al., 2018; Peciña & Zubieta, 2015). Placebos are not inert, but exert measurable biological effects.

The magnitude of the placebo response is not uniform across conditions. Conditions such as nausea or smoking seem to show relatively smaller placebo responses compared with insomnia or phobia (Krogsbøll, Hróbjartsson, & Gøtzsche, 2009). The placebo response in psychotropic drug trials has a relatively large effect size. Approximately 30% of patients in antidepressant and antipsychotic trials respond to placebo treatment (Furukawa et al., 2016; Leucht et al., 2018; Stein, Baldwin, Dolberg, Despiegel, & Bandelow, 2006; Walsh, Seidman, Sysko, & Gould, 2002). Two meta-analyses have shown that within-group pre-to-post effect size for placebo treatment ranges from 0.65 to 1.29 in anxiety disorders (Bandelow et al., 2015; De Vries, De Jonge, van den Heuvel, Turner, & Roest, 2016). These data demonstrate that the placebo response is an important and clinically relevant effect in psychiatry. However, it remains poorly understood.

In this paper, we argue that more research is needed into the placebo response in psychiatric medicine. We make the case that we need to understand this phenomenon for three broad reasons. First, we believe that improving our understanding of the placebo response within clinical trials will allow us to better evidence the efficacy of new treatments. Second, we feel that by understanding how to take advantage of placebo mechanisms operating in clinical settings, we will be able to maximise the effects of current treatments. Finally, exploring the biological mechanisms of placebo effects might reveal tractable targets for novel treatment development.

Although we focus on placebo in pharmacotherapeutic contexts, it should be noted that placebo responses also occur in psychotherapy. Furthermore, the factors leading to symptom improvement attributable to placebo may differ between pharmacotherapy and psychotherapy. For example, the quality of the clinician–patient interaction is not specific to drug efficacy and could be attributed to placebo, but this is potentially a factor more relevant to the efficacy of psychotherapy (Blease, 2018; Enck & Zipfel, 2019). Exploration of the placebo response in psychotherapy might improve our understanding of the specific mechanisms underlying its benefits, and potentially inform our understanding of the factors involved in placebo response in pharmacotherapy. A full discussion of these issues is outside the scope of the current paper, however, we refer the reader to a recent review in which this has been explored in detail (Enck & Zipfel, 2019).

## Improving ability to evidence efficacy of new treatments

In recent years, many pharmaceutical companies have ‘pulled out’ of neuroscience research, including into neuropsychiatric disorders. One of the major factors behind this is the late-stage failure of potential treatments to show efficacy in phase II or III clinical trials (Skripka-Serry, 2013). Potential neuropsychiatric drugs show a large amount of attrition from phase I trials to approval. Of the 60% of compounds that progress to phase II trials, a third will progress to phase III, but less than half of these will be reviewed by regulatory bodies and only 8.2% will be approved (McArthur, 2017).

One factor that contributes to this high attrition rate is the placebo response. The placebo response in antidepressant trials has previously been reported to be substantial and growing (Walsh et al., 2002). In antipsychotic trials, the magnitude of the placebo response has increased over the past 40 years, while the effect sizes of medication have remained stable (Agid et al., 2013; Leucht et al., 2017). The result is that the clinical trial as an assay exhibits reduced sensitivity to detect separation between active medication and placebo (Enck, Bingel, Schedlowski, & Rief, 2013). Interestingly, a 2016 meta-analysis and meta-regression showed that the placebo response rate in antidepressant trials increased from 1978 to 1991, but from 1991 it has remained constant at 35–40% (Furukawa et al., 2016). The meta-regression performed in this study showed that trials lasting longer than 4 weeks, multi-centre trials, and trials with flexible dosing regimens were all associated with increased placebo response rates (Furukawa et al., 2016). The important finding here was that once certain methodological parameters became constant between the years 1990 and 2000 (duration of 8 weeks, multi-centre trials made up over 90% of all studies, fixed dosing became more common) the placebo response rate also became constant. Although it is likely impossible and might even be unhelpful to eliminate placebo response (Whitlock, Woodward, & Alexander, 2019), this result suggests that we can standardise certain factors in trial design and thus control placebo response rate. By reducing the variability of placebo response rate trial to trial, we can ensure that clinical trials are properly powered to detect an effect of active medication and thereby reduce the likelihood of ‘failed trials’. However, it is probable that the important factors which need to be standardised will differ between diverse conditions. For instance, in antipsychotic trials it is increased sample size, shorter trial duration, shorter pre-trial washout, the rating scale used, studies outside the United States and shorter duration of illness that are associated with an increased placebo response rate (Leucht et al., 2018). We need to identify the important factors for all neuropsychiatric conditions and apply these insights in the design of psychotropic trials.

Consideration also needs to be given to the within-subject factors that increase placebo response rate. Placebo effects result from an interplay between prior expectations and subsequent learning (Ashar et al., 2017; Benedetti, Amanzio, Rosato, & Blanchard, 2011*a*, *b*). These mechanisms are known to be at play in clinical trials. For instance, the more active treatment arms there are in a trial, the higher the placebo response rate (Papakostas & Fava, 2009; Woods, Gueorguieva, Baker, & Makuch, 2005). This probably stems from an increased expectation from the patient that they will be randomised to receive an active medication. This is supported by the finding that the same drug produces larger effects in open trials compared with double-blind trials, i.e. when the patient is certain to receive active medication (Jensen et al., 2017; Rutherford et al., 2017). A patient's expectations will subsequently be updated through experience and learning (Ashar et al., 2017). For example, placebo analgesia is reduced if participants have experienced a previously ineffective analgesic treatment (Colloca & Benedetti, 2006; Kessner, Wiech, Forkmann, Ploner, & Bingel, 2013; Zunhammer et al., 2017). Such learning effects potentially confound crossover designs (Enck et al., 2013), and previous treatment experiences might influence a patient's expectations on entry to a clinical trial and their subsequent outcome (Benedetti, Carlino, & Piedimonte, 2016; Huneke & Baldwin, 2015). However, these possibilities are yet to be empirically tested, and the potential size of the effects is unknown. A simple first step would be to measure patients' expectations before and during clinical trials and include this parameter as a covariate in analysis of end-points (Benedetti et al., 2016). Another option to eliminate the influence of previous experience could be to prefer treatment-naïve patients, but it is currently unclear whether this reduces placebo response rate. It also remains unclear whether an individual who has responded to a placebo once is likely to do so again in future (Enck, Klosterhalfen, & Weimer, 2016). A full understanding of when, for whom, and to what degree placebo response can occur is needed.

Another suggestion to maximise assay sensitivity has been to measure placebo effect size through the inclusion of a ‘no treatment’ or ‘natural history’ control arm in clinical trials. In theory, patients in such a group would not be expected to improve, or if there was improvement then this would be the result of non-specific effects such as regression to the mean. Therefore, any difference between this control arm and the placebo arm would be due to a placebo effect. However, this design is not only ethically questionable (Enck et al., 2013) but is likely to be biased. A 2014 trial in patients with depression included such a ‘no-treatment’ arm, but the dropout rate in this arm was 40%, compared with 25% in the antidepressant arm and 10% in the placebo arm (Leuchter, Hunter, Tartter, & Cook, 2014). Such a large dropout rate will likely bias outcome measurements. One possibility to overcome this could be to use novel trial designs, such as a modified Zelen design, in which participants could be recruited to an observational study and a random sub-group then approached to participate in a clinical trial (Enck et al., 2013; Zelen, 1979). This would go some way to overcoming ethical issues and reduce the likelihood of dropouts. Such novel trial designs need to be tested, however, to ascertain their acceptability to potential participants, and to ensure that placebo response and effect of active medication can be accurately measured.

In summary, we need to understand which factors increase the chance for placebo response, for whom this occurs, and when. With this information, we could optimise trial designs to improve the chance of detecting efficacy of a novel treatment. We need to understand whether measuring expectations or using treatment-naïve patients would be beneficial. Finally, we need to test whether novel trial designs could improve assay sensitivity.

## Maximising effects of current treatments

It is accepted among physicians and psychiatrists that placebo mechanisms including the patient's expectations and previous experience of treatment can affect the effectiveness of psychotropic drugs in clinical practice. A survey of 87 physicians in Germany showed that more than 60% agreed that patient expectations and prior experience mediates the effectiveness of antidepressants (Kampermann, Nestoriuc, & Shedden-Mora, 2017). If this is true, it would follow that understanding how to ensure experiences are positive and expectations are maximised would allow us to optimise the effectiveness of our treatments.

There is indeed empirical evidence that placebo mechanisms can affect the effectiveness of interventions in the clinic. This can be demonstrated by the ‘open-hidden paradigm’. In these experiments, active medication is administered to a patient either in full view, or in a hidden fashion by a machine or through instructions that no medication is being given. Since the treatment is the same, the difference in effectiveness between the interventions is inferred to result from changes in the patient's belief and expectations (Wager & Atlas, 2015). A number of studies have shown that open administration of treatment in acute pain and Parkinson's disease is superior to hidden administration (Amanzio, Pollo, Maggi, & Benedetti, 2001; Atlas et al., 2012; Benedetti et al., 2003; Colloca, Lopiano, Lanotte, & Benedetti, 2004). This has also recently been demonstrated to be the case in social anxiety disorder. Patients openly given escitalopram improved with an effect size twice that of patients who received escitalopram but were told it was an ‘active placebo’ (*d* = 2.24 *v. d* = 1.13, respectively) (Faria et al., 2017). Furthermore, in patients with post-traumatic stress disorder, enhanced expectations of benefit was associated with higher likelihood of early response to sertraline, and improved outcomes after 10 weeks (Graham et al., 2018). The importance of expectations and beliefs for the effectiveness of treatments has led to the suggestion that we could develop interventions to improve pre-treatment expectations where they are particularly low (Enck et al., 2013). Such interventions and their effect on outcome have yet to be tested.

It has been argued that the doctor–patient relationship is key in activating beneficial placebo mechanisms in clinic (Thompson, Ritenbaugh, & Nichter, 2009), and perhaps we should be exploring this to understand how to maximise expectations. Although it would seemingly be clear that this should be the case, there is very little empirical supporting evidence. Some systematic review evidence shows that ‘clinician warmth’ and ‘listening’ are associated with patient satisfaction (Henry, Fuhrel-Forbis, Rogers, & Eggly, 2012), and that practitioners who are ‘warm and friendly’ are more effective than those who are ‘impersonal or uncertain’ for a range of conditions including hypertension, asthma and pain (Di Blasi, Harkness, Ernst, Georgiou, & Kleijnen, 2001). However, many studies included in these reviews were of poor quality and likely biased. Beyond this, there has been very little systematic exploration of how the doctor–patient interaction influences patient outcome, particularly in psychiatric medicine. We need more investigations that aim to understand whether the doctor–patient interaction matters, and if so, how doctors should interact with patients to maximise the benefits of any intervention.

Another important factor that influences treatment effects is adherence to medication. The more adherent a patient is, the more effective the treatment is likely to be. However, adherence with psychopharmacological treatments in those with severe mental illnesses is estimated to be only 40–50%, and a major contributor to poor medication adherence is the experience of side-effects (Velligan, Sajatovic, Hatch, Kramata, & Docherty, 2017). It is possible that placebo mechanisms could be utilised to improve adherence. The act of informing patients about possible side-effects of a medication engenders expectations that increase the chance the patient will report such side-effects (Neukirch & Colagiuri, 2015). This is an example of a ‘nocebo effect’: the experience of an adverse effect that is not attributable to the active ingredients of the treatment or therapy (Barsky, Saintfort, Rogers, & Borus, 2002; Petrie & Rief, 2019). Similar to how positive expectations and learning interact to produce placebo effects, it is thought that negative prior expectations and subsequent experience interact to produce nocebo effects (Petrie & Rief, 2019). For example, in a cohort of women receiving endocrine treatment for breast cancer, negative expectations at baseline increased the relative risk of side-effects over the course of 2 years, some of which were not attributable to the treatment (Nestoriuc et al., 2016). Additionally, changing the label of a placebo from branded to generic can reduce its effectiveness and increase reporting of side-effects in healthy volunteers, presumably because generic medicines are considered to be of ‘poorer quality’ (Colgan et al., 2015; Faasse, Cundy, Gamble, & Petrie, 2013; Petrie & Rief, 2019). Nocebo mechanisms are likely clinically relevant in the experience of side-effects and therefore adherence to medication. Theoretically it follows that to maximise current treatments we need to not only maximise placebo effects but also minimise the possibility of nocebo effects. Although it is necessary to fully inform patients of potential risks of treatment, framing this information positively, for example ‘90% of people will be unaffected’, reduces the chance a patient will report side-effects (Webster, Weinman, & Rubin, 2018). Further research is required to understand how best to minimise nocebo effects in psychiatric medicine while maintaining informed consent, and whether this improves adherence to medication.

Another placebo mechanism that could be exploited to improve medication adherence is learning through classical conditioning. If a medication is paired with an unconditioned stimulus, such as a green drink, for a number of administrations then the green drink will eventually induce effects similar to active medication on its own. Such classical conditioning paradigms have successfully induced placebo immunosuppression and placebo analgesia (Babel et al., 2017; Goebel et al., 2002). One study has shown that such mechanisms could also be important in neuropsychiatric conditions. Ninety-nine children with attention-deficit hyperactivity disorder aged 6–12 years old were randomly assigned to 8 weeks of treatment in one of three arms: reduced-dose + placebo, reduced-dose only, or treatment as usual. All were treated with an optimal dose of mixed amphetamine salt for 4 weeks, but in the reduced-dose + placebo arm, treatment was paired with a visually distinctive placebo capsule. At 4 weeks, the dose of mixed amphetamine salt was reduced by 50% in the reduced-dose and reduced-dose + placebo arms. The reduced-dose only group showed a significant worsening of symptoms by week 8, while the reduced-dose + placebo and treatment as usual groups did not differ in symptom severity (Sandler, Glesne, & Bodfish, 2010). There are potential sources of bias in this study, including that the children's parents were the severity raters and were not blinded to the intervention: nevertheless, the results suggest that it might be possible to reduce side-effect burden and thus improve treatment adherence through placebo mechanisms.

In summary, further understanding is required regarding how pre-treatment expectations might influence patient outcome in the psychiatric clinic, and whether interventions to improve expectations are beneficial. We need further studies into the doctor–patient relationship, and how doctors could best interact with patients to maximise treatment effects. Finally, we need to explore whether placebo mechanisms could be used to improve medication adherence. All of these could lead to changes in practice that maximise the effectiveness of our current treatments.

## Identification of novel treatment targets

As yet, research into the placebo effect has not led to identification of novel pharmacological targets in other fields. However, in psychiatry, it is possible that research into the placebo effect will directly lead to identification of tractable targets. It has been theorised that placebo effects are mediated by a ‘relaxation’ or reduction in negative emotions in anticipation that a distressing symptom might soon improve (Benedetti et al., 2011*a*, *b*; Flaten, Aslaksen, Lyby, & Bjørkedal, 2011). In psychiatry, the symptom targeted by treatment is often a patient's emotional state. Therefore, placebo mechanisms that act through changes in emotion might be directly clinically relevant. Placebo administration has indeed been shown capable of improving feelings of unpleasantness, disgust and negative mood through conditioning procedures or verbal suggestions (Glombiewski, Rheker, Wittkowksi, Rebstock, & Rief, 2019; Petrovic et al., 2005; Schienle, Ubel, Schongassner, Ille, & Scharmuller, 2014).

The question that follows is whether there are any neurobiological systems that could mediate the change in emotional state via placebo administration. There is a system that is common to placebo analgesia and placebo effects on emotion: the endogenous opioid system. Placebo-induced reductions of ‘unpleasantness’ are associated with increased rostral anterior cingulate cortex activity (Petrovic et al., 2005). This region is also known to be important in placebo analgesia (Atlas & Wager, 2014) and is a key node in the endogenous opioid system (Fields, 2004). Accumulating evidence suggests that the endogenous opioid system plays an important role in the experience of different affective states (Nummenmaa & Tuominen, 2018). It is therefore possible that, as is the case with placebo analgesia and opioid analgesics, recruitment of the endogenous opioid system in placebo effects on emotion would suggest that exogenous agents targeting this system could prove successful in treating affective symptoms. Recent evidence supports this argument. In a 2015 study, 35 depressed patients were scanned through positron emission tomography at baseline and following 1 week of a placebo treatment that was suggested would cause symptom improvement. This was followed by open-label antidepressant treatment for 10 weeks. The results showed that baseline *μ*-opioid binding potential in the nucleus accumbens, and degree of placebo-induced opioid release in nucleus accumbens, thalamus and subgenual anterior cingulate cortex, correlated with improvement in symptoms following antidepressant treatment (Pecina et al., 2015). Indeed, the endogenous opioid system is now being investigated as a possible therapeutic target in depression (Browne & Lucki, 2019).

Other neurobiological systems have been implicated in mediating placebo effects that might have relevance for placebo effects on emotion. For example, there is good evidence that the dopaminergic system is important in placebo effects. The ventral striatum, an important centre of dopaminergic neurotransmission, is reliably activated by placebo analgesia (Atlas & Wager, 2014) and degree of dopamine release in the nucleus accumbens explains 25% of the variance of placebo analgesic effects in healthy volunteers (Scott et al., 2008). Additionally, placebo effects in Parkinson's disease are mediated by the dopaminergic system (De La Fuente-Fernandez, 2001; Lidstone et al., 2010). Another example is the endocannabinoid system, which appears to mediate placebo analgesic effects conditioned by non-opioid analgesics such as ketorolac (Benedetti et al., 2011). Finally, hypothalamic–pituitary–adrenal axis activity and the cholecystokinin system have been linked to nocebo hyperalgesia (Benedetti, Amanzio, Vighetti, & Asteggiano, 2006). All of these systems are relevant to psychiatric symptoms and phenomena such as anxiety, anhedonia and psychosis. Indeed, both placebo analgesia and nocebo hyperalgesia involve activity in brain regions that process the affective component of pain, suggesting that the neurobiological systems that drive these responses might have direct effects on emotion (Atlas & Wager, 2014; Kong et al., 2008). However, it is presently unknown whether non-opioid systems are important in placebo effects on emotion. Understanding which of these other neurobiological systems are important might reveal additional tractable therapeutic targets for psychiatric medicine.

If we can identify biomarkers related to placebo effects under psychiatric conditions, then we might identify important neurotransmitter systems involved in symptom improvement and additional novel treatment targets. There has been some research exploring biomarkers of placebo response in depression and in anxiety, but these studies have often been re-analyses of data collected for other purposes and have only attempted to measure biomarkers at the end of treatment (Faria et al., 2012; Mayberg et al., 2002). To fully understand the neurobiological systems underpinning placebo effects in patients, we need to carry out prospective studies in which the primary aim is to identify placebo mechanisms. We also need longitudinal studies with measures at multiple time-points to understand how brain activity or other biomarkers change during the course of a placebo treatment. This might allow us to identify the activity, systems and time-points that are most important for therapeutic effects at the end of treatment.

## Conclusion

In this paper, we have argued that more research is needed into the placebo response in psychiatry. We have shown that understanding the factors that cause placebo responses in clinical trials, and whether there are novel ways to measure this effect, is necessary for improving clinical trial design to better evidence the efficacy of new treatments. This is important as many novel treatments fail in the late stages of development, so any improvements in this area could allow more treatments to gain approval. Furthermore, we have shown that improved understanding of how placebo mechanisms including expectation and learning operate in the clinic, might allow us to maximise the effectiveness of our current treatment arsenal. Finally, early evidence in the field of depression has shown that research into the placebo response could lead to the identification of novel treatment targets in neuropsychiatric disease. For these reasons, it is vital that we pursue research into the placebo response.
